# Volume XXV

**Published:** 1885-08

**Authors:** 


### Volume XXV.

The present number opens the twenty-fifth volume of the Journal. The high standing and character which the Journal has heretofore maintained, and its well-established reputation, constitute the assurances we have to offer for its future management. The JOURNAL must be the reflex of professional activity and research in this section of the State. We can only be the impartial exponents of that activity. We renew the request previously made, that our pages are open to the profession for the publication of clinical reports, papers on subjects of professional interest, communications on subjects relating to medical science, and, indeed, whatever will add to the progress of medicine. It is intended to make this volume of greater value than any of its predecessors. An able corps of collaborators has been secured, who will cull from the current medical literature the more valuable abstracts for the use of the practitioner, and furnish the latest advances made in medical subjects. We look for a largely increased circulation as the results of our labors. The last volume was more widely distributed than that of any previous year. We expect a like increase for the coming year.

---

It is announced in the secular press that Prof. Mason, who for many years has filled the chair of Physiology in the Buffalo Medical College, has tendered his resignation. Thus another ot the old regime has given up the work. Only one remains, Prof. Rochester, of those whose professional reputation has maintained the institution in the position it has heretofore held. Dr. Julius Pohlman has been appointed lecturer on Physiology for the coming session. The appointment is an excellent one. We know Dr. Pohlman to be an earnest worker, and an enthusiast in scientific research. The position will be ably filled; and that, too, by a member of the profession in this city, an exception to the rule adopted of late years in filling vacancies in its faculty. Both the College and the profession are to be congratulated on the change which has been made, and we anticipate a large measure of success for the new lecturer.

